# The Advantages and the Dangers of Intrauterine Injections

**Published:** 1882-07

**Authors:** Charles W. Pilgrim

**Affiliations:** Asst. Physician, Asylum for Insane Criminals, Auburn, N. Y.; Late House Physician, Bellevue Hospital, N. Y.


*FOR CONTENTS OF THIS NUMBER SEE PAGE 504.*

*THE*

### Independent Practitioner.

VOL. III.——*July, 1882.*——No. 7.

### ORIGINAL COMMUNICATIONS.

*We are not responsible for the opinions expressed by contributors.*

ARTICLE I.

### THE ADVANTAGES AND THE DANGERS OF INTRA-UTERINE INJECTIONS.

BY CHARLES W. PILGRIM, M.D.
Asst. Physician, Asylum for Insane Criminals, Auburn, N. Y.
Late House Physician, Bellevue Hospital, N. Y.

Cohnstein in his classical account of intra-uterine injections ascribes their first use to the ancients more than two thousand years ago, but Freund in his able criticism published in the *Deutsche Klinik* shows that he has fallen into the error of supposing that the word uterus had the same meaning with them as with us, whereas, it is well known that they included both the vagina and uterus under this term, and what he considers intra-uterine injections were, in all probability, merely vaginal. Their earliest authenticated use was by Lisfranc and Vidal de Cassis, the latter being the first to institute experiments upon the cadaver bearing upon this subject. The use of the intra-uterine injection is a therapeutical measure which has many advocates on account of its great utility, and many opponents as well because of its manifest dangers. The following case, which occurred in my practice while interne in Bellevue Hospital, illustrates one of the results to be feared when instruments are used which are not entirely suited to the purpose. A. B., æt. twenty, was confined for the first time two days before admission. She had never had any miscarriages, had always been regular in menstruation, which began when she was fourteen years of age. Her history did not indicate that her labor had been in any way unnatural. On admission, October 17th, her temperature was 99° F. She complained of slight pain in the region of the uterus, and there was a slight lochial discharge, for which carbolized vaginal injections were ordered morning and evening. She was in a fair condition in other respects. This state continued until 6 A.M. October 19th, when she was seized with a chill, after which her temperature rose to 104° F. Quinia, in antipyretic doses, was ordered, and, as it was noticed on the previous evening that the lochial discharge was much more offensive and high-colored than usual, it was thought advisable to wash out the uterus. A "fountain syringe" was filled with water and suspended about three feet above the level of the bed. To this *an ordinary female catheter* was attached, as nothing more suitable was at hand.

On passing the finger into the vagina the temperature of the parts was found to be very much increased; the os was high up and the cervix dilated enough to admit the end of the forefinger. The catheter was then passed along the forefinger of the left hand until it reached the os externum, through which it readily passed. The current of water being turned on, it entered the uterus, but could not be discovered flowing out over the perineum. When the syringe (or rubber bag) was about half emptied the woman commenced to cry out with pain, and the catheter was immediately withdrawn. Her sufferings seemed to be intense, and in a few minutes her pulse became small and rapid, her abdomen began to swell, her face became deadly pale, her extremities grew cold, and her whole frame trembled as though shaken by a violent chill. Magendie's solution of morphia, M. x., was given subcutaneously, and hot bottles were applied to the feet. A half ounce of whiskey was also given by mouth. The injection was given at 11 o'clock, and at 11.30 she had another violent chill, and at 12 M. her temperature had risen to 106° F. Her body was sponged with equal parts of ice-water and alcohol, for the purpose of reducing the temperature, and at 1 P.M. it had fallen to 103¾° F., at 3.30 P.M. to 101¼° F. and at 7 P.M. it was only 100° F. She passed a comparatively quiet night, and, as the injection was not repeated, she went on to convalescence without a recurrence of any of the unfavorable symptoms. It is quite evident that the above-mentioned phenomena were due to the retention of the water, and the consequent forcible contraction of the uterus upon its foreign contents, just as the agonizing pains in some cases of dysmenorrhœa are merely due to the irritation and contractions produced by the retained blood. Although this condition is one of no great danger to the patient, the symptoms are very alarming in appearance, and I must confess that I have never since repeated the procedure without a good deal of care and some misgivings. Besides the result illustrated by my own case, either of the following more serious ones may occur :

1. The fluid may pass through the Fallopian tubes into the peritoneal cavity, resulting in peritonitis and death. It is true that the experiments of Vidal, Hennig, Klemm, Scanzoni and others, upon the cadaver, show that it is only with extreme difficulty that fluid can be made to enter the tubes ; but it must be remembered that these experiments cannot be of very great value on account of the essential differences between the dead and living bodies. In the former there is neither nervous irritability nor muscular contractility, both of which, it is not improbable, may become important agents when under the stimulus of the injection, by causing the cervix to contract spasmodically about the nozzle of the syringe, thus retaining the irritant and setting up uterine action sufficient to pump the fluid onward into the tubes.

Dr. Whitehead, in a paper published in the *British Medical Journal*, even suggests that capillary or ciliary action may be sufficient to carry the fluid into the peritoneal cavity. But, even granting that the experiments referred to have proved that such a disastrous result is *improbable*, the cases reported by Van Haselberg, Barnes and Kerns, all of which were verified by autopsies, show conclusively that it is *possible*, and is especially liable to occur when disease has destroyed sphincteric action at the os internum and metro-salpingian orifices and rendered the tubes unusually patent ; when any undue force is used in injecting the fluid, or when there exists any abnormality, such as a flexion or a very narrow cervix, which would prevent its ready return into the vagina.

2. When there is any laceration of the uterine mucous membrane, sudden death may be caused by the entrance of air into the veins. Such an accident occurred in the practice of Bessems, who, upon making an autopsy, found "air-bubbles in the vena cava and heart." A similar case which occurred in the Charity Hospital of St. Louis is also reported by Dr. Warner. And

3. The fluid, especially if it be of a styptic character, I will not say caustic, may cause metritis, and the inflammation may spread, producing metro-peritonitis, which it may be very difficult to control. Indeed, this result may be caused by water alone when it is injected in a manner to cause forcible dilatation of the uterus.

Although all of the preceding accidents have occurred and are always liable to occur in careless and inexperienced hands, by the exercise of skill and care intra-uterine injections may be made play an important part in the therapy of gynæcology. They, of course, should never be used when there is an existing flexion of the uterus, nor just before or after menstruation, nor when pelvic peritonitis or cellulitis has recently existed. If the physician who desires to resort to this method can eliminate the above objections, and will then see that the fluid can readily escape by using a tent before the injection unless the os internum be dilated, and will inject the solution (which at first should be mild and of a temperature of 85° or 90° F.), very gently through *a double canula syringe*, from which all air has been expelled, he will find it a plan of treatment both valuable and safe.

